# Quantitative Modeling of *Escherichia coli* Chemotactic Motion in Environments Varying in Space and Time

**DOI:** 10.1371/journal.pcbi.1000735

**Published:** 2010-04-08

**Authors:** Lili Jiang, Qi Ouyang, Yuhai Tu

**Affiliations:** 1 Center for Theoretical Biology and School of Physics, Peking University, Beijing, China; 2 The State Key Laboratory for Artificial Microstructures and Mesoscopic Physics, School of Physics, Peking University, Beijing, China; 3 IBM T. J. Watson Research Center, Yorktown Heights, New York, United States of America; University of Illinois at Urbana-Champaign, United States of America

## Abstract

*Escherichia coli* chemotactic motion in spatiotemporally varying environments is studied by using a computational model based on a coarse-grained description of the intracellular signaling pathway dynamics. We find that the cell's chemotaxis drift velocity *v_d_* is a constant in an exponential attractant concentration gradient [*L*]∝exp(*Gx*). *v_d_* depends linearly on the exponential gradient *G* before it saturates when *G* is larger than a critical value *G_C_*. We find that *G_C_* is determined by the intracellular adaptation rate *k_R_* with a simple scaling law: 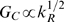. The linear dependence of *v_d_* on *G* = *d*(ln[*L*])/*dx* directly demonstrates *E. coli*'s ability in sensing the derivative of the logarithmic attractant concentration. The existence of the limiting gradient *G_C_* and its scaling with *k_R_* are explained by the underlying intracellular adaptation dynamics and the flagellar motor response characteristics. For individual cells, we find that the overall average run length in an exponential gradient is longer than that in a homogeneous environment, which is caused by the constant kinase activity shift (decrease). The forward runs (up the gradient) are longer than the backward runs, as expected; and depending on the exact gradient, the (shorter) backward runs can be comparable to runs in a spatially homogeneous environment, consistent with previous experiments. In (spatial) ligand gradients that also vary in time, the chemotaxis motion is damped as the frequency *ω* of the time-varying spatial gradient becomes faster than a critical value *ω_c_*, which is controlled by the cell's chemotaxis adaptation rate *k_R_*. Finally, our model, with no adjustable parameters, agrees quantitatively with the classical capillary assay experiments where the attractant concentration changes both in space and time. Our model can thus be used to study *E. coli* chemotaxis behavior in arbitrary spatiotemporally varying environments. Further experiments are suggested to test some of the model predictions.

## Author Summary

A computational model, based on a coarse-grained description of the cell's underlying chemotaxis signaling pathway dynamics, is used to study *Escherichia coli* chemotactic motion in realistic environments that change in both space and time. We find that in an exponential attractant gradient, *E. coli* cells swim (randomly) toward higher attractant concentrations with a constant chemotactic drift velocity (CDV) that is proportional to the exponential gradient. In contrast, CDV continuously decreases in a linear gradient. These findings demonstrate that *E. coli* senses and responds to the relative gradient of the ligand concentration, instead of the gradient itself. The intracellular sensory adaptation rate does not affect the chemotactic motion directly; however, it sets a maximum relative ligand gradient beyond which CDV saturates. In time-varying environments, the *E. coli*'s chemotactic motion is damped when the spatial gradient varies (in time) faster than a critical frequency determined by the adaptation rate. The run-length statistics of individual cells are studied and found to be consistent with previous experimental measurements. Finally, simulations of our model, with no adjustable parameters, agree quantitatively with the classical capillary assay in which the attractant concentration changes both in space and time. Our model can thus be used to predict and study *E. coli* chemotaxis behavior in arbitrary spatiotemporally varying environment.

## Introduction

**Figure 1:**
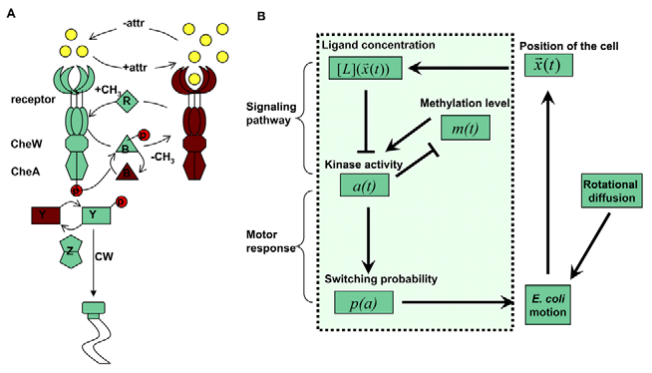
Illustrations of the E. coli chemotaxis pathway and the SPECS (Signaling Pathway-based *E. coli* Chemotaxis Simulator) model. (A) The *E. coli* chemotaxis signal transduction pathway. The MCP complex receptor-CheW-CheA is the sensor and can be active (green) or inactive (dark brown). Binding of attractant molecules (yellow) decreases the probability of the receptor to be active. Once activated, the histidine kinase CheA quickly autophosphorylates and then transfers the phosphate group to CheY. CheY-p, the response regulator, diffuses to the flagellar motor and modulates its switching probability between CW and CCW rotations. CheZ, the CheY-p phosphatase, serves as the sink of the signal. Adaptation of the system is carried out by methylation and demethylation of the receptor, which are facilitated by the enzymes, CheR and CheB-p, respectively. (B) Flow chart of the SPECS model (reproduced from Figure 3 in [21]). The input for the signaling pathway (inside the box) is the instantaneous ligand concentration 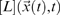. The internal signaling pathway dynamics is described at the coarse-grained level by the interactions between the average receptor methylation level 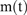 and the kinase activity 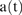, which eventually determines the switching probability of the flagellar motor 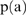. The switching probability is then used to determine the cell motion (run or tumble), and direction of motion during run fluctuates due to rotational diffusion. The motion of the cell leads it to a new location with a new ligand concentration for the cell and the whole simulation process continues.

Bacterial chemotaxis is one of the most studied model systems for two-component signal transduction in biology [1]. In *Escherichia coli*, the relevant proteins and their interactions in the chemotaxis signaling pathway have been studied over the past decades and a more or less complete qualitative picture of chemotaxis signal transduction has emerged (Figure 1A). It is now known [2] that external chemical signals are sensed by membrane-bound chemoreceptors called methyl-accepting chemotaxis proteins (MCP), which form a functional complex with two types of cytoplasmic proteins: the adaptor protein CheW and the histidine kinase CheA. Upon binding to an attractant (repellent) ligand molecule, the receptor suppresses (enhances) the autophosphorylation activity of the attached CheA, and transduces the external chemical signal to inside the cell. The histidine kinase CheA, once phosphorylated, quickly transfers its phosphate group to the two downstream response regulator proteins CheY and CheB [3]. The small protein CheY-phosphate (CheY-p), before it gets dephosphorylated by the phosphatase enzyme CheZ, can diffuse from the receptor complex to the flagellar motor. CheY-p can bind to FliM proteins of the flagellar motor, increasing the probability of changing its rotation from counterclockwise (CCW) to clockwise (CW), which in turn causes the motion of the *E. coli* cell to change from run to tumble. After a brief tumble, the cell runs again in a new random direction. The directed motion of bacterial chemotaxis is achieved when the run length is longer in a favorable direction [1].

Significant progress has been made in several key areas towards quantitative understanding of the *E. coli* chemotaxis signaling pathway. First, through experiments and modeling, it is now well established that the high sensitivity of the *E. coli* chemotactic response [4],[5],[6] is partly caused by the cooperative interaction between neighboring MCP complexes [7],[8],[9],[10] within the polar receptor cluster. Another important feature of the *E. coli* chemotaxis signaling pathway is its ability to adapt to a wide range of environments by slow methylation and demethylation of the MCP receptors at four specific residues. Two enzymes are involved in adaptation: CheR adds methyl groups to the chemoreceptor, while CheB-p removes them [3]. Both of these processes depend on the receptor kinase activity, and this feedback mechanism is believed to be responsible for the near perfect adaptation of the system [11],[12],[13],[14], which maintains the kinase activity within the sensitive range of the motor. A general model framework was recently developed to describe the adaptation kinetics and receptor cooperativity, and all previous experiments with time-varying signals can be explained consistently within this model [15]. Finally, the response of the *E. coli* flagellar motor to intracellular CheY-p level was measured quantitatively by Cluzel et al [16] at the single cell level. The dose-response curve has a high Hill coefficient, possibly caused by cooperative interactions between the FliM proteins in the FliM ring.

As pioneered by Dennis Bray [10],[17], computer modeling has been used in studying bacterial chemotaxis motion [18],[19],[20],[21]. With improved quantitative understanding of the chemotaxis signaling pathway, up-to-date knowledge of the key pathway components can be integrated to form a system-level model of the signaling network to quantitatively study various chemotaxis behaviors. In this study, we used a coarse-grained Signaling Pathway-based *E. coli* Chemotaxis Simulator (SPECS, an acronym introduced here for convenience) model to study chemotaxis behaviors in a series of environments with increasing spatiotemporal complexity. We originally developed the SPECS model to explain the recent microfluidics experiments with stationary linear gradients in [21]. Here, we focused on using this model to study *E. coli* chemotaxis motion in spatiotemporally-varying environments and to understand how the chemotaxis motion is controlled by the cell's internal molecular signaling processes, in particular its adaptation dynamics. Quantitative comparisons with the classical capillary assay [22],[23], where the attractant concentration changes both in space and time, were made to test and verify the model. We argue that the SPECS model can be used to predict quantitatively the motion of *E. coli* cells in any given spatiotemporally varying chemical field, such as in the natural environment.

## Methods

Here, we briefly describe SPECS, a parsimonious model first introduced in [21] that contains the minimum essential features of the *E. coli* chemotaxis pathway without including all the detailed elements and reactions of the entire network. To represent the system-level dynamics of the signaling pathway, we use a coarse-grained model in which the chemoreceptor is represented by its average kinase activity 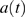 and its average methylation level 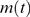 at time *t*. The external environment at time *t* is given by the ligand concentration 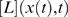 at the physical location 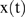 of the cell. The receptor ligand binding affects the kinase activity at a short time scale, while adaptation occurs through receptor methylation at a much longer time scale. The kinase activity regulates the probability (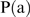) that the flagellar motor switches between CCW and CW states, thus controlling the tumble or run motion of the cell. As the cell moves, the ligand concentration 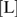 can change both directly due to temporal variation in 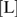 and indirectly because of the cell motion in environments with spatial-inhomogeneous ligand concentration. A flow chart of the simulation scheme is shown in Figure 1B. Quantitative details of our model are explained below.

### Chemotaxis signaling pathway dynamics

Following Tu et al. [15], each functional MCP receptor complex can be either in the active or the inactive state; these states are separated by a free energy difference, 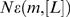, where 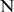 is the number of the responding receptor dimers in the complex. The ligand-receptor binding time 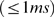, estimated from the measured ligand-receptor dissociation constant [24] and the diffusion limited on-rate, is much shorter than the receptor methylation time scale 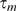 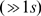. The measured CheA auto-phosphorylation time (∼0.025s) [25] is also much shorter than 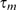. Therefore, the kinase activity can be determined by the quasi-equilibrium approximation:
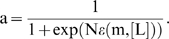
(1)Using the Monod-Wyman-Changeux allosteric model to describe the receptor cooperativity [26],[27], the free energy difference can be written as:
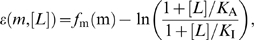
(2)where 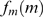 is the methylation level-dependent free energy difference; 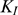 and 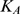 are the dissociation constants of the ligand to the inactive and the active receptor, respectively. Quantitatively, for MeAsp, which is the chemo-attractant studied here, we use the parameters 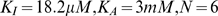 determined by fitting the pathway model to the *in vivo* FRET data [28]. The free energy contribution due to methylation of the receptor is taken to be linear in 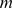: 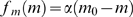 as used in [15] and supported by recent experiments [29]. The parameters 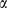 and 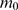 can be estimated from the dose-response data [6],[30] of the *cheRcheB* mutants with different methylation levels; for MeAsp, they are roughly 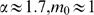.

The kinetics of the methylation level can be described by the dynamic equation [15]:
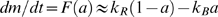
(3)The general methylation rate function 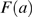 is expressed by a linear approximation with 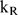 and 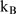 as the linear rates for methylation and demethylation processes. The simple form is based on the assumption that CheR only methylates inactive receptors and CheB-p only demethylates active receptors, which are required to achieve near perfect adaptation in the kinase activity [11],[13],[14],[31]. More complicated Michaelis-Menten equations can be used [32], but they do not affect the results here as the system (pathway) normally operates in the linear range. For simplicity, we take 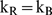 to fix the steady state activity 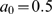; another value 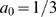 was used without affecting the results. The methylation rates can be estimated by the adaptation time from experiments with step function stimuli [4]; for MeAsp, we use 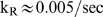. The dependence of the chemotaxis motion on 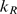 is studied in this paper.

### Run and tumble motion

A simple phenomenological model is used here to model the *E. coli* cell motion. Let 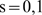 represent the tumble and run states of the cell. For the time period 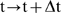, a cell switches from state 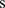 to state 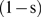 with probability 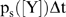. The response curve measured by Cluzel et al [16] determined the ratio between the two probability rates for one flagellar motor (see Supporting Information (SI) Figure S1 for details on effects of multiple flagella):
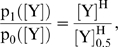
(4)with 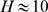 and 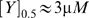. We assume the tumble time is roughly constant (independent of 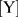) by setting 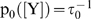, where 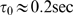. is the average duration of the tumble state. Correspondingly, the probability rate to switch from the run state to the tumble state is:
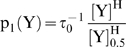
(5)In our simulation, 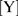 is assumed to be proportional to the kinase activity: 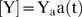 without considering the nonlinear dependence [33] (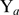 is defined later in this paragraph). This linear approximation is justified by the relatively small range of activity variation in our study. Including the CheY-p dephosphorylation dynamics explicitly with dephosphorylation time 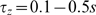 did not significantly change the results (see Figure S1). Spatial effects are neglected as the diffusion time for CheY-p across the cell length is short 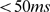 [34] and the CheY-p level was measured to be spatially uniform in wt *E. coli* cells [35]. In steady state, 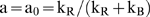 and the average run time is 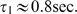. Therefore, 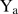 is set by 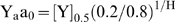. After a tumbling episode, a new run direction is chosen randomly with the run velocity 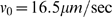 [36]. In our simulations, a small time step 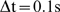 is chosen to resolve the average tumbling time.

### Rotational diffusion

As first pointed out by Berg and Brown [37], one important factor in chemotaxis is the rotational diffusion of the cell due to the Brownian fluctuation of the medium. This can be simply captured by adding a small Gaussian random angle 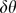 to the direction of the velocity in every run time step 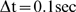.:
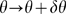
(6)The amplitude of this rotational diffusion angle 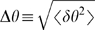 is roughly 10° as estimated by the fact that it takes ∼10sec. for the cell to lose its original direction of motion (i.e., turn more than 90°) by pure rotational diffusion.

### Boundary effect

For the boundary condition, we assume that when a cell swims to a wall, it swim along the wall for some time (1–5 sec.) before swimming away [21],[38]. The boundary condition can affect the cell distribution near the wall, but should not strongly affect the overall behavior of the cell distribution in the bulk.

## Results

The SPECS model was used to investigate *E. coli* chemotaxis behaviors (for individual cells and at the population-level) for a series of ligand profiles with increasing spatial and temporal complexity. Our model revealed the key dynamics of the microscopic control circuit responsible for these behaviors and predicted novel responses to spatio-temporally varying environments, which can be tested by future experiments.

### *E. coli* chemotactic motion in stationary ligand gradients: logarithmic sensing and its microscopic mechanism

#### Constant chemotactic drift velocity in exponential ligand gradients

**Figure 2:**
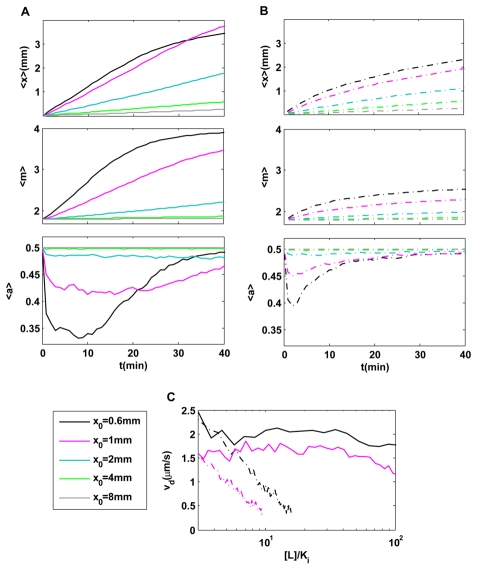
Comparison of chemotactic motions in exponential and linear ligand profiles. (A) Cell motion and intracellular dynamics in exponential ligand concentration profiles: 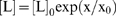. (B) Cell motion and intracellular dynamics in linear ligand profiles: 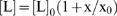. In both (A) & (B), the dynamics of the (population) averaged position (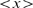); the average receptor methylation level (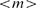) and the average kinase activity 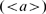 are shown for different decay lengths 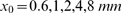. 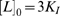. The population-averaged position increases linearly with time until the methylation level reaches saturation in exponential profiles; while it slows down continuously in the linear profiles. After a transient decrease, the kinase activity stays roughly constant in exponential profiles, while it varies continuously recovering to its pre-stimulus level 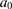 in linear profiles. (C) Direct comparison of instantaneous velocities between exponential (solid lines) and linear (dotted lines) profiles for 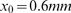 (black); 1.0 mm (purple). Within the chemosensitivity range 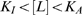, the instantaneous chemotaxis drift velocity is constant in exponential profiles, while it decreases continuously with [L] in the linear ligand profiles.

One central question in chemotactic motility is whether the drift velocity of the cells is determined by the gradient (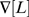) or the relative gradient (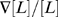) of the ligand concentration 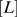. We addressed this question by studying cell motion in two types of stationary ligand spatial profiles, linear and exponential, in which the gradient or the relative gradient of the ligand concentration was kept constant, respectively. For an exponential attractant concentration profile 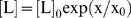, the relative gradient 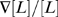 (or equivalently the gradient of the logarithmic concentration 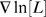) is a constant vector along the x-direction with magnitude 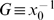. The response to pure temporal exponential gradient was first studied experimentally by Block et al [39]. Recently, exponential gradient sensing was studied theoretically [15] and from an optimization point of view [19]. Here, by using the SPECS model, we simulated the motion of a population typically consisting of 1000 individuals in different exponential ligand profiles (solid lines, Figure 2A). In comparison, cell motions in linear gradients: 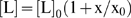 was also studied (dotted lines, Figure 2B). We calculated the dynamics of the average cell position, the average methylation level, and the average activity of the cells for different values of 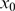.

In exponential gradients, the average cell position increases linearly with time, leading to a constant chemotaxis drift velocity 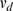, before it saturates at a later time. The molecular mechanism for this behavior becomes evident by inspecting the average methylation level of the cells. Initially, as a cell moves up the gradient, its receptor methylation level increases with time to balance the effect of the increasing ligand concentration. This adaptation mechanism maintains the kinase activity within the sensitive range of the flagellar motor and therefore maintains a constant chemotactic drift velocity. Only when the receptor methylation level approaches its maximum value (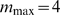), the chemotactic motion slows because the cell can no longer adapt to higher ligand concentrations, which can be seen in Figure 2A for the case of 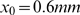 for 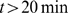. In contrast, the drift velocity decreases continuously in linear gradients long before the methylation level reaches its maximum (Figure 2B). The difference in drift velocities in exponential and linear gradients originates from the different behaviors in their average kinase activities. In an exponential gradient, the kinase activity shifts to a new steady-state value lower than the perfectly adapted value 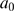 in the absence of any gradient (Figure 2A). The activity shift 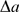 measures the kinase response of the cell. The constant kinase responses in exponential gradients lead to constant drift velocities. In a linear gradient, the kinase activity, after an initial fast decrease, continuously recovers towards 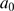 without reaching a steady state, as shown in Figure 2B. The decreasing kinase response in a linear gradient leads to a decreasing drift velocity (Figure 2B). In Figure 2C, plots of instantaneous drift velocity as a function of ligand concentration for both exponential (solid line) and linear (dotted line) gradients show explicitly that cells move with constant drift velocities in exponential gradients while they slow down in linear gradients as they move to regions with higher ligand concentrations.

The range of ligand concentrations over which the drift velocity remains constant in an exponential gradient is spanned by the two dissociation constants 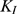 and 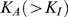 for inactive and active receptors respectively. From Eq. (2), the free energy contribution from ligand binding, 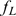, can be expressed as: 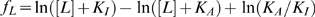. Within the range 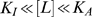, 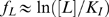. This logarithmic dependence of ligand concentration in the free energy leads to a constant kinase activity shift in response to an exponential temporal gradient [15],[39] and eventually results to a constant drift velocity proportional to the gradient of the logarithmic ligand concentration, i.e., the logarithmic sensing behavior. For MeAsp, this range (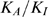) is over 2 decades as shown in [6],[28]. In general, a constant activity shift can be obtained by setting the rate of change in activity free energy to be a constant: 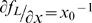, resulting to the required ligand profile:
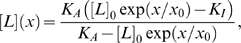
(7)where the constant 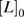 sets the scale for the ligand concentration. Thus, the required ligand profile is exponential: 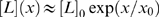 as long as 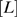 is within the range set by 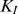 and 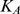: 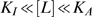. Recently, Vladimirov et al [20] studied the dependence of drift velocity on gradient shape and adaptation rate with a much smaller range (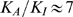) assumed for Asp. A constant drift velocity was reported in [20] for a ligand profile that is quantitatively different from the exponential gradient shown here. This discrepancy is likely caused by the smaller 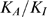 ratio used in [20]. In addition, an uncontrolled approximation for changes in ligand free energy 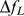 was used in [20] to obtain the specific form of ligand profile for constant activity. Linearizing the exponential spatial dependence in the exact solution given in Eq. (7) would lead to a similar (but not identical) spatial gradient form as reported in [20]. However, such linearization is only valid for a limited range of space 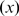, which is much smaller than the length scale 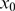 of the ligand profile.

The logarithmic sensing behavior, i.e., constant drift velocities in exponential gradients, predicted here from the model can be directly tested by measuring *E. coli* chemotaxis motion in stationary exponential attractant gradients, which is yet to be achieved experimentally. Recently, we used the SPECS model to simulate *E. coli* chemotactic motions in a finite channel with different linear ligand profiles. The quantitative agreement with microfluidics experiments [21] indirectly supported the notion that *E. coli* senses the relative change of ligand concentration and verified the validity of the SPECS model.

#### Adaptation rate controls the critical exponential gradient and the saturation (maximum) chemotaxis velocity

**Figure 3:**
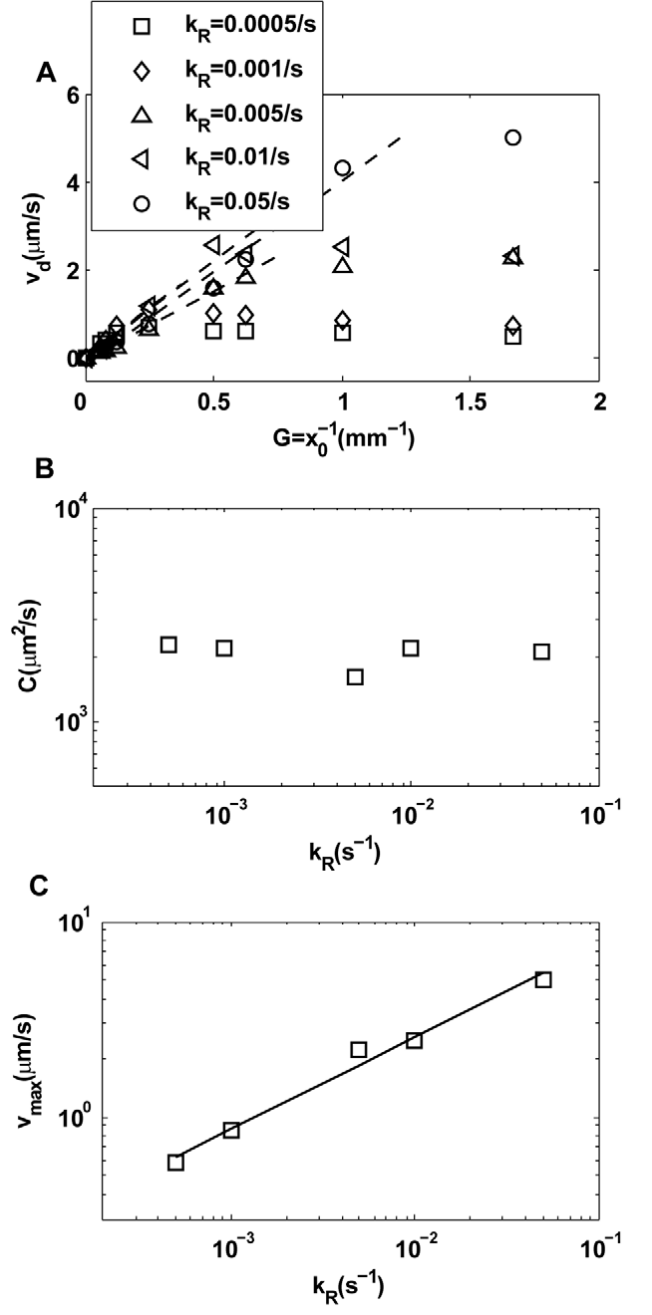
Dependence of chemotaxis motion on the adaptation rate. (A) The chemotaxis drift velocity 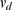 for different exponential gradient 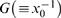. Different symbols represent different adaptation rates 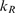. Note that 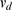 first increases linearly (dashed line) with 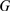 before reaching a saturation velocity at a critical gradient 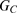,. We can fit 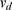 with: 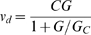, in which 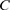 is the chemotaxis motility constant given by the linear fitting coefficient and the saturation drift velocity is 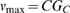. The dependences of 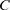 and 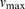 on 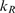 are shown in (B) and (C) respectively. For the range of 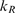 we studied, we found that 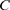 is roughly independent of 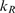 and 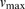 depends on 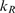 with a simple scaling relation: 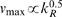.

Within the chemosensitivity range (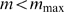), the average cell position 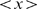 increases linearly with time in exponential gradients, resulting in a constant chemotaxis velocity 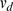: 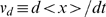. The dependence of 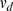 on the exponential gradient 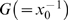 is determined for different values of 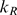 (with fixed 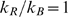), which correspond to different adaptation rates. As shown in Figure 3A, the drift velocity is linearly proportional to the gradient of the logarithmic ligand concentration, 
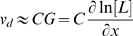
, for 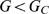 where 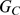 is a critical gradient beyond which 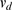 saturates. The linear proportional constant 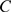 defines the chemotaxis motility of *E. coli* cells and has the dimension of a diffusion constant with its scale set by 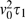, where 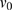 and 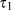 are the average run velocity and run time, respectively. The dimensionless motility constant 
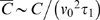
 is directly controlled by the sensitivity of the cell motion to relative ligand concentration changes, and is proportional to the signal amplification at both the receptor and the motor levels.

For 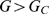, 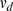 saturates and becomes independent of 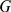. This transition depends on the adaptation rate characterized by 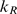 (Figure 3A). Phenomenologically, the chemotaxis velocity for the full range of gradients can be approximately written as:
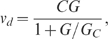
(8)and the maximum (saturation) chemotaxis velocity is simply:
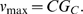
(9)Fitting the drift velocities (Figure 3A) with Eq. (8) for different adaptation rates 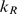, we can quantify the dependence of chemotaxis motion on the adaptation rate. As shown in Figure 3B, the motility constant 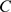 is roughly independent of 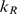, but the maximum chemotaxis velocity 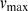 is controlled by the adaptation rate with a scaling dependence 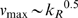. So from Eq. 9, we have: 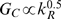.

From Eq. 8, it is clear that 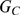 represents the maximum exponential gradient to which a cell can respond normally (linearly). The scaling dependence of 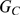 on the adaptation rate 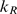 can be understood by the internal signaling pathway dynamics. For a temporal exponential concentration ramp with ramp rate 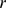, it was shown in [15] that the kinase activity shifts by a constant 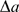 which is proportional to 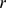 and the adaptation time 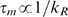. For a cell moving with a drift velocity 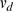 in a spatial exponential gradient 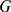, the effective average ramp rate it experiences is 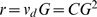. Therefore, the kinase activity shift can be obtained as in [15]: 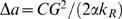. The flagellar motor responds to the kinase activity within a narrow fixed range of size 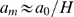, where 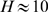 is the Hill coefficient of the motor response curve [16]. For a given 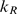, the adaptation rate is not fast enough to keep the kinase activity within this operational range of the flagellar motor for a very steep gradient, and this critical gradient 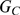 is therefore determined by 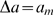, which leads to the observed scaling relation: 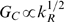.

Taken together, a simple coherent picture of *E. coli* chemotaxis motion emerges from our modeling studies. Within the chemo-sensitive regime (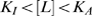) the chemotaxis drift velocity is linearly proportional to the relative gradient of the concentration. The signal amplification of the underlying pathway increases the chemotaxis motility; and the internal adaptation rate determines the range of this linear response and the maximum drift velocity as summarized in Eq. (8). A few interesting results come directly from this general picture. In a linear gradient 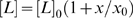, the dynamics of the average *E. coli* position 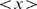 can be studied by using Eq. (8) and assuming 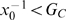: 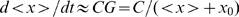, which leads to an analytical solution: 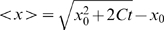. This analytical solution agrees with our simulation results (Figure 2B). It shows explicitly that the chemotaxis drift motion in a linear gradient is sub-linear with a continuously decreasing velocity and 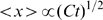 at long times when 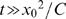. For a given exponential gradient 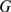, because of the weak dependence of the motility 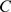 on 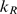 within the range of adaptation rates we studied, the dependence of the chemotaxis drift velocity on 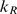 does not show a pronounced maximum at a particular adaptation rate as reported previously [20]. Instead, the same (near-optimum) chemotaxis velocity is reached, provided that the adaptation rate 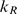 is larger than a minimum rate 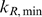 to keep the cell within the linear response regime, i.e., 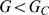. From the dependence of 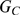 on 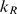, we can obtain the dependence of 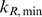 on 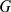 : 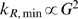. Of course, extremely fast adaptation with adaptation times approaching the average run time would drastically decrease the drift velocity as the cell loses its ability to distinguish forward runs from backward runs.

#### Chemotactic motion of individual cells: statistics of the forward and backward run lengths

**Figure 4:**
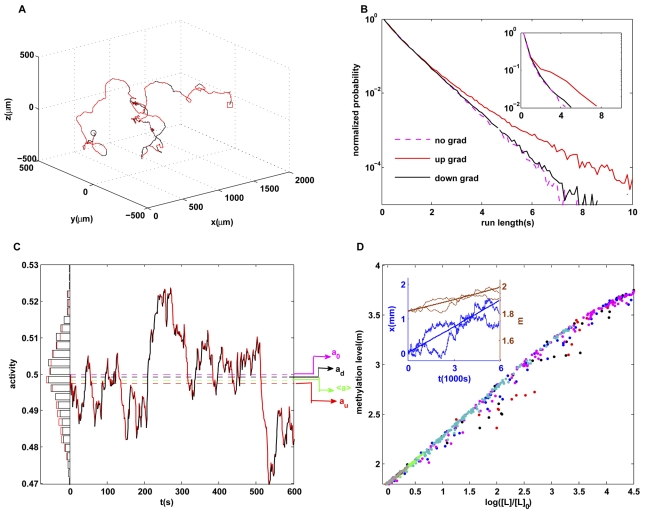
Single cell behaviors in exponential attractant gradients. (A) Trajectory of a cell for 10 min for 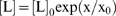 with 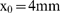. The random walk motion is biased towards the gradient (arrow). The forward runs up the gradient are in red, and the backward runs down the gradient are in black. (B) Run length distribution for forward runs (red), backward runs (black) in the presence of an exponential gradient and without a gradient (purple). The backward run length distribution is close to the run length distribution in the absence of a gradient, similar to the experimental results from Berg and Brown [37] as reproduced in the inset. (C) Distributions of cell kinase activity for forward (red) and backward (black) runs. A time series of the activity 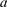 of a single cell is shown: forward is in red and backward is in black. The average activity for backward (black dashed line) is closer to the adapted activity (purple dashed line, 0.5) compared with the average activity for forward (red dashed line). (D) Methylation level of different individual cells at different times and in different exponential gradients (represented by color symbols as in Figure 2) all increase with logarithmic ligand concentration along a universal line, despite large temporal fluctuations in methylation levels and positions for (two) individual cells as shown in the inset.

In their early work, Berg and Brown first observed long tracks of *E. coli* motion and measured the distributions of runs by using a 3D tracking microscope [37]. From the characteristics of the longer runs up the gradient than down the gradient, they first established that bacterial chemotaxis results from longer runs toward attractants. They also found that the distribution of runs down the gradient is similar to the run length distribution in the absence of ligand concentration gradient. This peculiar observation prompted the question of whether the cell only responds to upward the gradient while ignoring the downward gradient. Here, we addressed this question by using our model to study the characteristics of the chemotactic motion of individual cells. We showed the motion of one cell in an exponential ligand gradient profile (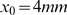) (Figure 4A) and the distributions of the run time in the forward and backward directions (Figure 4B). The average forward run time is longer than the average backward run time, with the distribution of backward run time close to the distribution in the absence of attractant gradient. Our model results are consistent with the experimental observations [37] (Figure 4B inset) without invoking different sensing mechanisms for the downward and upward gradients.

The microscopic mechanism for the run length distributions can be studied with our model. We found that after some initial transient time, the kinase activity fluctuated around a constant average value 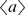. This steady state activity value 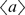 was found to be lower than 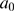, the steady state activity in the absence of ligand gradient, by an amount that depends on the steepness of the gradient (Figure 4C). This result can be explained as follows. As the cell drifts up the exponential ligand gradient with a constant velocity, the average ligand concentration the cell experiences at time *t* grows exponentially with *t*. For an exponential temporal stimulus, it was first shown experimentally by Block et al [40] that the kinase activity of the cell shifts to a constant value lower than its adapted value. As explained recently in [15], this constant shift in activity is caused by the balance of the exponentially increasing ligand concentration with the linearly increasing methylation level of the receptors. The methylation level versus the logarithmic ligand concentration for individual cells at different times for different exponential gradients collapse onto a universal curve (Figure 4D). The logarithmic external ligand concentration is closely tracked by the receptor methylation level despite the large temporal fluctuations in both these quantities (Figure 4D). Due to the fact that 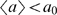, the overall average of all the run times (both up and down the gradient) is longer than that in the absence of gradient. This also explains the larger cell diffusion constant in the presence of exponential attractant gradients (Figure S2). However, during an individual run down the gradient, the decreasing ligand concentration increases the kinase activity as methylation is too slow to react in the typical run time scale. The opposite is true for the upward run. Indeed, as we examined the kinase activity statistics during the upward and the downward runs separately (Figure 4C), we found that the average kinase activity during downward runs 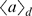 was larger than that of the upward runs 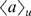: 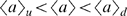. Therefore 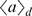 can approach 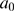 (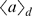 can be smaller or bigger than 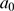, depending on the methylation rate and ligand gradient) while 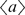 and 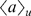 are always smaller than 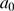.

Analytically, the activity shift can be obtained by using the results from the last subsection and following the analysis in [15]: 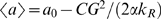. The deviation of 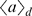 and 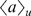 from 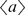 can be estimated due to slow adaptation during average run time 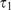: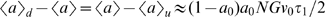, which gives: 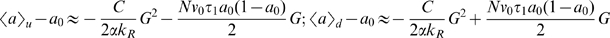. These analytical expressions show that while 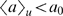 is always true, 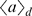
can be larger, smaller, or equal to 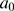 depending on the exponential gradient 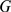 (See Figure S3 in SI for demonstration of all these cases). Quantitatively, the run time distributions depend on the details of the gradient, e.g., the ligand concentration in [37] probably has a Gaussian profile rather than a pure exponential form.

### *E. coli* chemotactic motion in oscillating spatial gradients: damped responses at high frequencies

**Figure 5:**
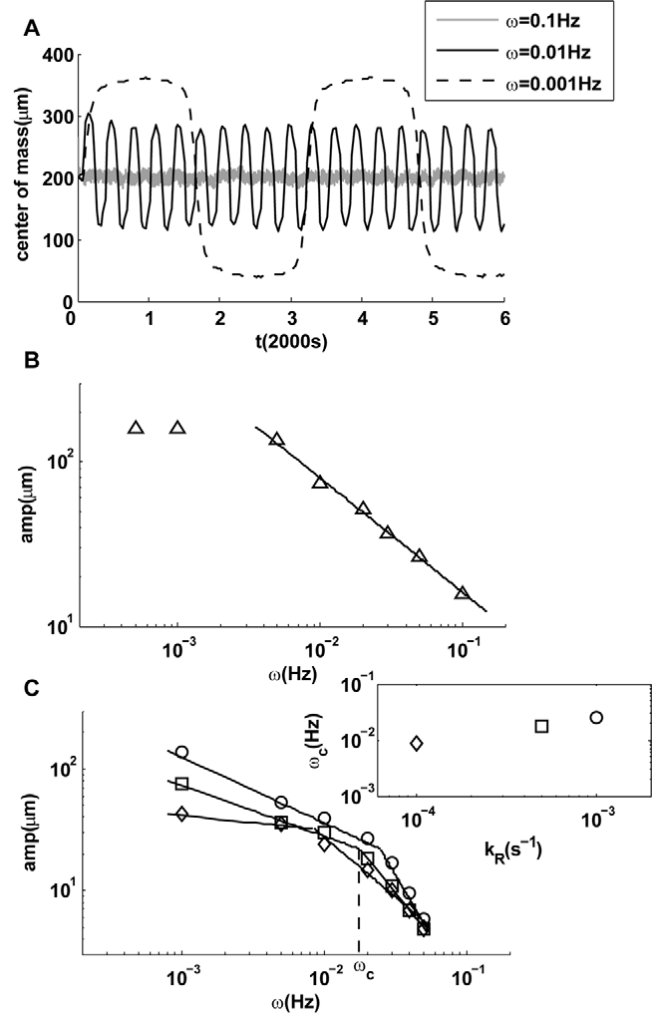
Responses to oscillating linear gradient. (A) Time dependence of the average positions of cells for three oscillatory gradients, all with the same amplitude but different frequencies (ω). The responses have the same frequencies as their driving signals, but the response amplitude decreases with the driving frequency. (B) The amplitude of the response decreases with frequency ω. The cross-over at low frequency (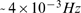) is caused by boundary effects. (C) Upon decreasing adaptation rate, a transition to a steeper decay of the amplitude appears at frequencies higher than a transition frequency 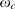 within the range of frequencies studied. Three cases with smaller values of 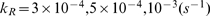 are shown, and the dependence of 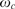 on the adaptation rate 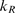 is shown in the inset of (C).

In the natural environment, chemical signals not only vary in space, they also fluctuate in time. The fluctuation of a chemical signal (ligand concentration) sensed by a moving cell can be caused by: 1) randomness in the cell motion, i.e., the run-tumble motion and the rotational diffusion of the cell; and 2) temporal variation of the environment itself. Here, we investigated the effects of the latter due to ligand (spatial) gradients that also vary with time. In particular, based on the feasibility of future experimental tests of our predictions, we studied the case that *E. coli* swims in a finite channel of length 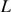 where the attractant concentration 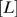 is linear inside the channel 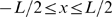 with a slope that oscillates in time with a frequency 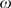:
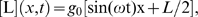
(10)with a fixed maximum ligand spatial gradient 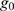. 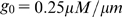 was used in this study. We simulated the motion of cells and their dependence on the frequency 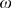 in a channel with 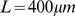, the same geometry as used in our previous study of the stationary linear gradient [21]. To separate the effects of the time-varying gradient from those caused by the fast time variations due to run-tumble transitions (∼1 s) and rotational diffusion (∼10 s), we studied the dependence of cell motion on ligand concentration oscillation with relatively low frequency 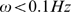. We found that the average position (center of mass) of the *E. coli* cells oscillated with the same periodicity as the ligand concentration (Figure 5A). The amplitude of the response depends on the frequency (Figure 5B). For spatial gradients changing with very low frequencies, the response amplitude becomes comparable to the size of the channel and stays almost constant independent of 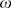 due to the boundary effects. For higher frequencies, the amplitude the average cell motion decreases with 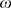. This dependence of cell motion on the frequency of the gradient can be understood by studying the mean-field dynamics of the average position of the cell 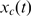 by assuming an instantaneous response to the (logarithmic) ligand gradient:
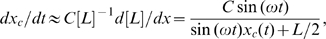
(11)where 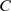 is the motility constant defined in the last section. For high frequency, the amplitude of the cell motion is much less than the channel size, 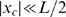, and the above equation can be solved approximately to obtain:
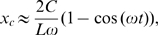
(12)which shows that the response amplitude decreases with frequency as 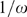, consistent with our simulation results (Figure 5B). Equation (12) breaks down and the amplitude saturates at low frequencies 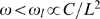, determined by the finite channel size 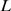 (Figure 5B). Quantitatively, the full Eq. (11) does not yield to any simple scaling dependence of response amplitude on 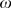.

How do the adaptation dynamics affect the cell's response to time-varying gradients? We investigated this question by varying 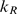 (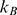 was co-varied to keep 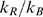 fixed). We found that for smaller values of 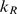, a transition frequency 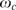 appears within the frequency range 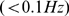 studied here (Figure 3C). For frequencies higher than 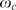, the decay in response amplitude became significantly steeper than for frequencies below 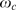. The transition frequency 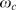 is determined by the adaptation rate, with faster rates resulting in higher 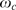 (Figure 5C, inset). This transition to faster decay in response amplitude is likely caused by the finite adaptation (response) time 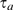 of the underlying signaling system, which leads to the dependence of the instantaneous drift velocity on the relative gradients in its past with a exponentially decaying function with time scale 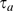:
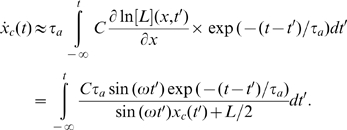



If the time-dependent term in the denominator of the integrant of the above equation is neglected for small amplitudes of cell motion, we can estimate the amplitude of cell motion at high frequencies: 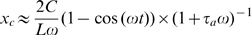, which has a similar time-dependence as in Eq. (12) but with an extra factor 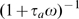 due to the finite adaptation time. For high frequencies 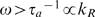, this extra factor causes the response amplitude to decay with an extra factor 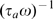, consistent with our simulation results (Figure 5C).

The responses of *E. coli* to pure temporal oscillatory signals have been studied experimentally [39], theoretically [15], and within the framework of information theory [41]. However, for an *E. coli* cell moving in a time-varying spatial gradient, its signaling pathway dynamics becomes much more complex as the signal (ligand concentration) changes due to both its temporal and spatial variations convoluted by the cell motion, which is in turn determined by the pathway dynamics. The interplay between spatial and temporal signals coupled to cell motion can lead to rich cellular behaviors, which we have just started to explore. The quantitative dependence of cell motion on 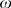 and 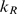 depends on the details of the ligand spatial profile and a simple analytical form is not available. However, the damped chemotaxis motion in environments with high-frequency gradients should be generally true due to the finite adaptation time of the cell. This frequency-dependent chemotaxis behavior can be tested by future experiments in spatial gradients that also change with time with tunable frequencies.

### Complex spatial-temporal ligand profile: quantitative simulation of the classical capillary assay

**Figure 6:**
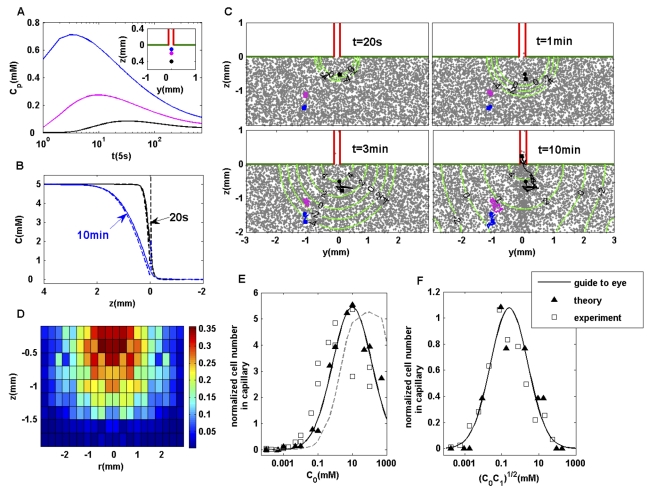
Quantitative simulation of the classical capillary assay and comparison with experiments. (A) Time-dependent ligand concentrations at three different positions in the suspension pool (see inset) from directly solving the ligand diffusion equation. The ligand concentration at a given position peaks at a given time, depending on its location. 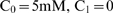. (B) The exact ligand profile (solid line) along the center line of the capillary at different times, in comparison with the asymptotic solutions (dashed lines) by Furtelle and Berg [42]. (C) Cell density in the rectangular coordinate is shown together with the contours of the logarithmic ligand concentration (in 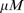) at different times. Three individual cell trajectories (starting from circles and ending at squares) are shown. Only the black cell ends in the capillary. (D) Probability distribution of the original positions of cells that end in the capillary. For a cell originally located at position 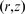, the probability of it ending in the capillary at a later time (45 min), 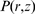, is shown. 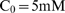, 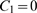. (E) Concentration-response curve for the capillary assay. The average number of bacteria in the capillary after 45–50 min subtracted by the number of bacteria in the capillary in the absence of attractant is defined as the response (ordinate). The results from our model with the exact ligand profile are labeled by solid symbols (fitted by a solid line). They agree well with the experimental measurements (hollow squares) of Mesibov et al [22],[23]. The results from using the asymptotic ligand profile by Furtelle and Berg are shown by the dashed lines. (F) Response curve for capillary assay with 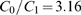. The solid symbols (fitted with a solid line) represent the model, and the hollow symbols represent the experimental results [22] (both collected at 60 min).

The capillary assay is an ingenious experimental method developed more than a century ago by W. Pfeffer and later perfected by J. Adler's group to study bacterial chemotaxis [22],[23]. A capillary tube containing a solution of attractant is inserted into a liquid medium (the pool) containing bacteria. A gradient of the attractant is subsequently developed due to diffusion and bacterial cells swim into the tube following the gradient. The number of bacteria entering the capillary is counted at a given time (45–60 min), as a measure of the cells' chemosensitivity. This method is still in use today because of its simplicity and also because the spatiotemporally varying attractant profile mimics the realistic situation of attractant released from a stationary source. Here, we modeled the responses of cells in the capillary assay and quantitatively compared the results with the experimental measurements. The time-dependent attractant concentration was determined by solving the diffusion equations:
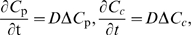
(13)where C_c_ and C_p_ stand for the ligand concentrations in the capillary and the suspension pool respectively. 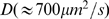 is the diffusion coefficient of the attractant ligand in water. Using the cylindrical symmetry of the geometry, the ligand concentration was solved in cylindrical coordinates with the boundary conditions:
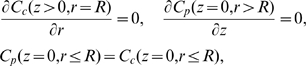
(14)where 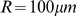 is the radius of the capillary tube. The initial conditions at 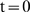 are that the ligand concentrations in the capillary and the suspension pool are C_0_ and C_1_ respectively: 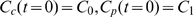. The time-dependent concentration profiles are shown in Figure 6A. Starting from being 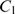 at 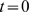, the ligand concentration at a particular position in the pool peaks at a given time depending on its location (Figure 6A, inset). Furtelle and Berg [42] calculated the attractant profile by solving the diffusion equations asymptotically away from the mouth of the capillary (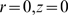), and their analytical asymptotic solution is shown together with our exact numerical solution in Figure 6B at different times along the center line of the capillary tube (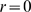). The two solutions show remarkable agreement except for near the mouth of the capillary, where the Furtelle and Berg solution breaks down. Later, we show this inaccuracy near the capillary mouth can cause large differences in computing the result of a capillary assay.

From the spatial-temporal profile of the attractant, cell motion can be calculated by using SPECS. We considered the bacterial cells started randomly in a region of 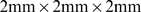 around the capillary mouth inside the pool (Figure 6C). The probability 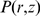 of a cell at an original position 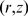 that eventually ends in the capillary at 45min was calculated (Figure 6D). Even cells originally far away from the mouth of the capillary can enter it, with 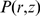 decreasing with both z and r.

Finally, we calculated the chemotactic responses in capillary assay for different values of 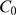 and/or 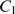 and compared the results directly with the experiments by Mesibov et al [22],[23]. The results showed that the number of bacteria accumulating in the capillary 45 min after the capillary is inserted is a function of 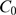 (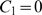) for both the experiments and our simulation (Figure 6E). The results from our model, with no adjustable parameters, agree quantitatively with the experiments. The dashed line in Figure 6E represents the results of our cell motility simulation by using the Furtelle and Berg solution for the ligand concentration. Evidently, even though the Furtelle and Berg solution is accurate away from the capillary mouth, its inaccuracy near the capillary mouth changes the results significantly. The accuracy of the ligand profile near the mouth of the capillary is important because cells that eventually enter the capillary need to pass the mouth. In another set of experiments by Mesibov et al [22], both 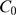 and 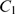 are changed while keeping their ratio fixed at 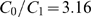. The sensitivity curves are plotted as the number of bacteria accumulating in the capillary after one hour versus 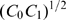 (Figure 6F). The simulation results (filled triangles) showed quantitative agreement with the experiments [17], further verifying our model. Qualitatively, the shape of the capillary assay response can be understood as the chemotaxis response (sensitivity) is small for either very large or very small ligand concentrations. Quantitatively, the peak response concentration is larger than the center of the chemoreceptor sensitivity region [6] due to the fast decay of the ligand concentration from inside the tube toward the pool (Figure 6A).

## Discussion

In this paper, a coherent picture of how *E. coli* chemotaxis motion depends on the spatio-temporally varying chemical environment and its intracellular signaling dynamics has emerged from our modeling study. The chemotaxis drift velocity 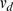 is mainly determined by three factors: 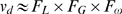, where 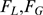, and 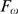 depend on the ligand concentration 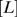, the gradient of the logarithmic concentration 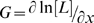, and the frequency 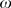 of the temporal-variation of 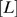 respectively. For ligand concentrations within a wide (chemosensitive) range 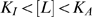 (as focused on in this paper), the concentration-dependent factor 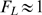, and 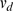 depend linearly on the gradient of the logarithmic concentration 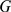 until it saturates at a high relative gradient 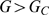 as demonstrated by the second factor 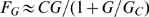. For a ligand gradient that varies with high frequency 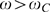, 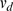 is damped by the third factor 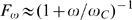 due to the finite adaptation time 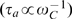 in the underlying signaling pathway. We found that the saturation gradient 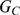 is controlled by the adaptation rate 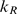, but the motility constant 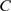 is not (although weak dependence on 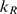 cannot be completely ruled out). The nontrivial scaling dependence 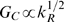, observed in our simulation, can be explained analytically by the dynamics of the internal signaling pathway and the narrow range of kinase activity over which the flagellar motor can response.

Calibrated quantitatively by the most up-to-date *in vivo* FRET experiments, our model (SPECS) captures the essential characteristics of the underlying signaling network, in particular the receptor-receptor cooperativity and the near-perfect adaptation kinetics, within a simple unified mathematical description. We described the internal state of a cell at the coarse-grained (cellular) level without modeling the details of individual signaling molecules as used in other simulation methods such as StochSim [17], AgentCell [18], and E. solo [10], which are particularly suited to studying noise in the intracellular signaling process. This coarse-grained approach, similar to that used in RapidCell [20], greatly reduces the computational requirements for the simulation. For example, the SPECS model allows us to simulate *E. coli* populations of 10^3^–10^4^ cells in a linear ligand concentration profile and 10^2^–10^3^ cells in a capillary assay *in real time* with a standard desktop computer (Matlab code available upon request); and the simulation results agree quantitatively with both the recent microfluidics experiments and the classical capillary assay measurements, without any fitting parameters. Predictions, such as bacterial chemotactic responses in exponential ligand profiles and oscillatory ligand gradients, are made with our model and can be tested by future experiments. Indeed, the SPECS model can be used to predict *E. coli* chemotaxis motions in arbitrary spatial-temporal varying environments efficiently and accurately.

Perhaps equally important as predicting cellular behaviors, the SPECS model, which captures the essential features of the underlying pathway, enables us to understand these behaviors based on the key intracellular signaling dynamics, some of which can be difficult to study directly by experimental methods. For example, the constant drift velocity in an exponential ligand profile was found to be caused by a constant shift in the average kinase activity, which is maintained by a linearly increasing mean methylation level in balancing the exponentially increasing ligand concentration. At the individual cell level, this constant activity shift is also shown to be responsible for the intriguing observation that the average backward run time in an exponential gradient is similar to the average run time in the absence of a gradient, while the forward run time is much longer.

The SPECS model can be used to study various noise effects as well. The effect of the cell-to-cell variability for chemotaxis behavior in a linear gradient in a closed channel was studied by choosing (from a broad distribution) a random value for the internal parameters such as *N*, methylation rate constants, and swimming velocity in each individual cell. We found that although individual cells now behaved differently, at the population level, the average steady state behavior, such as cell density, remained almost the same (see Figure S4) except for a slight change near the boundary for the case with run velocity variation. The main source of (external) temporal noise for the cell's chemotactic sensory system comes from the run and tumble motion of the cell. Even in a smooth spatial ligand gradient, the randomness in the cell motion can lead to large temporal fluctuations in the input (ligand concentration) to the *E. coli*'s chemotactic sensory system. This source of external noise was included in our model. The effects of fluctuations in intracellular signaling remain to be examined. By adding noise to our pathway model, it would be interesting to see whether and how the internal signaling noise affects the population level behavior.

The model framework described here lays the foundations for modeling cellular motility behavior based on the relevant underlying signaling pathway dynamics without describing the details of the individual signaling molecules. The current model can be extended in several directions to study many other interesting chemotaxis phenomena. For example, the interaction between the cells and the liquid-solid surface were oversimplified in this paper. Cells are known to turn with a preferred handedness when they run into a surface [43] and can remain near the surface for a long time [38] before they finally escape. It would be interesting to see how different “boundary conditions” affect the overall behavior of cells. In its natural environment, a cell must make decisions in the presence of multiple, sometimes conflicting cues. Our model can be extended to include integration between different chemotactic signals [26] and applied to study bacterial motion in the presence of multiple stimuli gradients. The same chemotaxis pathway seems to be able to sense and react to other non-chemical stimuli, such as temperature [44] and osmotic pressure [45]. Our model can be modified to study the responses of cells to these non-chemical stimuli by incorporating the dependence of various (kinetic and energetic) biochemical parameters on the strength of these external stimuli. Recently, we carried out such extensions to study the microscopic mechanism of precision-sensing in *E. coli* thermotaxis [46]. Finally, the chemo-attractant (MeAsp) considered in this paper is non-metabolizable and its concentration gradient is formed independent of the cell population. In other cases, such as in swarm plate experiments [47], the attractant gradient is generated by consumption of the nutrient, which is also the chemo-attractant. In addition, cells can communicate by emitting chemo-attractants [48]. The consumption and generation of the attractant, together with cell division, need to be incorporated into our model to understand complex pattern formations in different swarm plate experiments. We are currently pursuing some of these directions.

## Supporting Information

Figure S1 Effects of CheY-p dephosphorylation and multiple motors.
(0.06 MB PDF)

Figure S2 Chemotactic drift velocity and diffusion constant in exponential ligand concentration gradients.
(0.06 MB PDF)

Figure S3 Single cell behavior in different exponential gradients.
(0.04 MB PDF)

Figure S4 Effects of cell-to-cell variability.
(0.06 MB PDF)
