# Supplementary material for: Quantitative Modeling of Escherichia coli Chemotactic Motion in Environments Varying in Space and Time
Source: PLoS Comput Biol. 2010 Apr 8;6(4):e1000735. doi: 10.1371/journal.pcbi.1000735 (PMC2851563; doi:10.1371/journal.pcbi.1000735)
Supplement: Figure S1 — Effects of CheY-p dephosphorylation and multiple motors. (0.06 MB PDF) [file pcbi.1000735.s001.pdf]

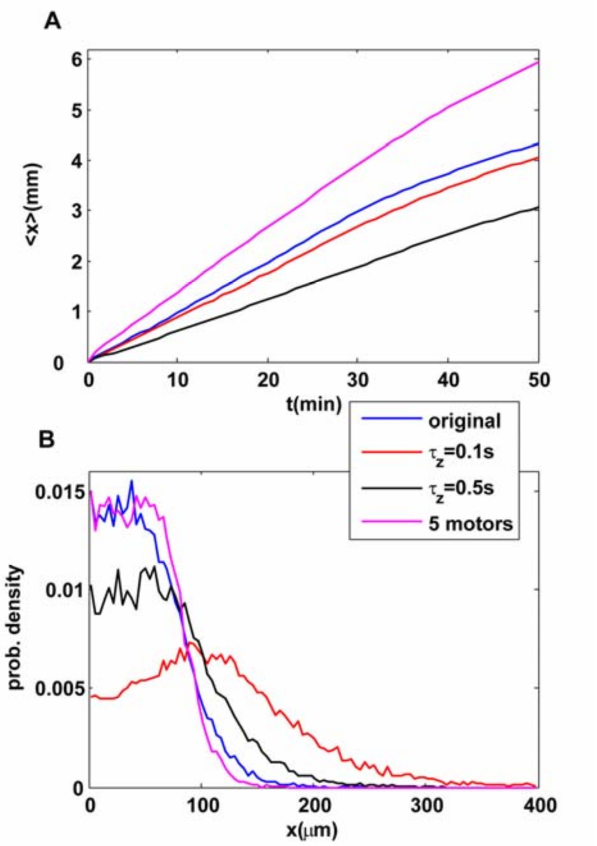

Figure S1 Effects of CheY-p dephosphorylation and multiple motors. The dynamics of CheY-p can be derived as $d[Y]/dt = -([Y] - Y_a a(t))/\tau_z$, where τ_Z is the time scale of dephosphorylation by CheZ. Here we consider *E. coli* has five motors which can independently switch from "run" to "tumble". The majority of all the five motors determine the final state of the cell. We checked the effects of CheY-p dephosphorylation and multiple motors both in infinite exponential $x_0 = 1mm$ (A) and boundaried linear gradient (B). These two effects can only quantitatively affect our results here but should not change our qualitative conclusions.
