# Supplementary material for: Quantitative Modeling of Escherichia coli Chemotactic Motion in Environments Varying in Space and Time
Source: PLoS Comput Biol. 2010 Apr 8;6(4):e1000735. doi: 10.1371/journal.pcbi.1000735 (PMC2851563; doi:10.1371/journal.pcbi.1000735)
Supplement: Figure S2 — Chemotactic drift velocity and diffusion constant in exponential ligand concentration gradients. (0.06 MB PDF) [file pcbi.1000735.s002.pdf]

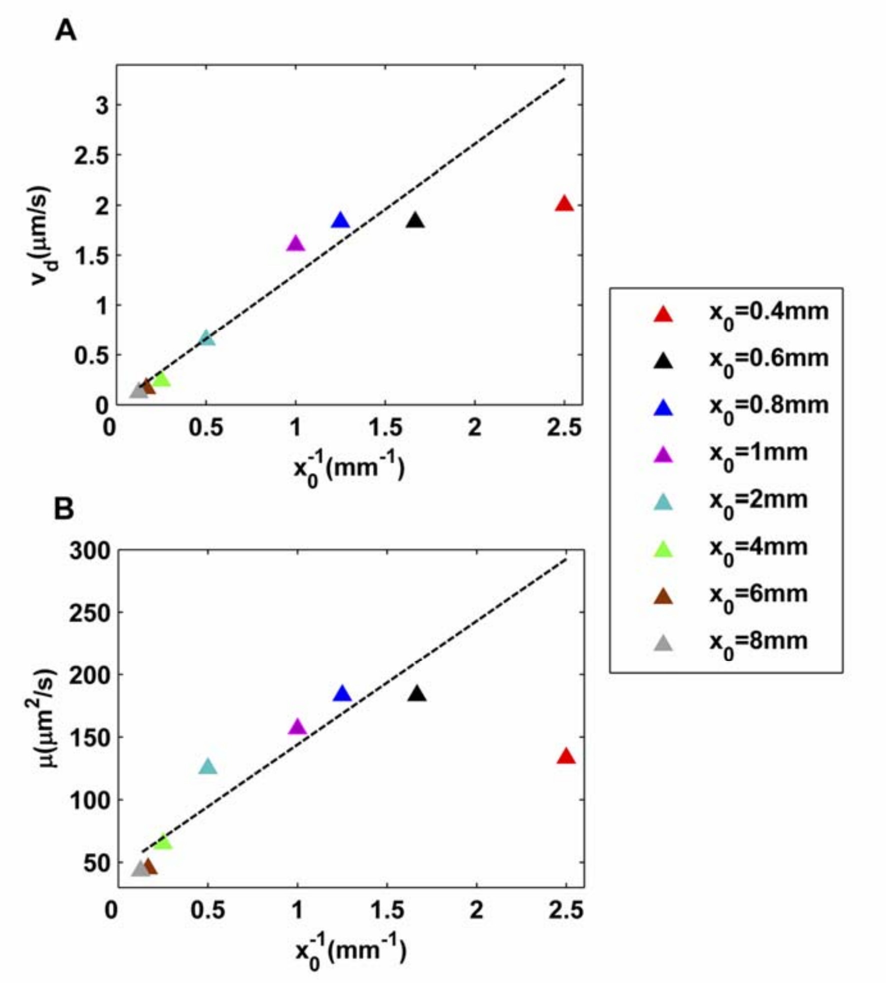

Figure S2 Chemotactic drift velocity and diffusion constant in exponential ligand concentration gradients. In exponential gradients both the center of mass $<x>$ and the mean standard deviation of cell locations $<x^2>$ increase linearly with time. So we plotted drift velocity $v_d = d<x>/dt$ and diffusion constant $\mu = d<x^2>/dt$ at different gradient G. They have the same transition gradient G_C which depends on the adaptation rate as explained in the text.
