# Supplementary material for: Quantitative Modeling of Escherichia coli Chemotactic Motion in Environments Varying in Space and Time
Source: PLoS Comput Biol. 2010 Apr 8;6(4):e1000735. doi: 10.1371/journal.pcbi.1000735 (PMC2851563; doi:10.1371/journal.pcbi.1000735)
Supplement: Figure S3 — Single cell behavior in different exponential gradients. (0.04 MB PDF) [file pcbi.1000735.s003.pdf]

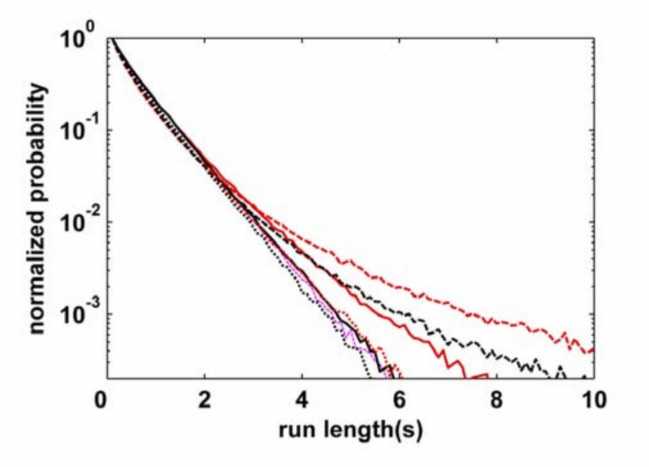

Figure S3 Single cell behaviors in exponential gradient. The same as Figure 4B but we added the statistics for $x_0 = 2mm$ (dashed line) and $x_0 = 8mm$ (dotted line). Quantitatively, the run time distributions depend on the details of the gradient. The experimental results probably correspond to a Gaussian profile.
