# Supplementary material for: Quantitative Modeling of Escherichia coli Chemotactic Motion in Environments Varying in Space and Time
Source: PLoS Comput Biol. 2010 Apr 8;6(4):e1000735. doi: 10.1371/journal.pcbi.1000735 (PMC2851563; doi:10.1371/journal.pcbi.1000735)
Supplement: Figure S4 — Effects of cell-to-cell variability. (0.06 MB PDF) [file pcbi.1000735.s004.pdf]

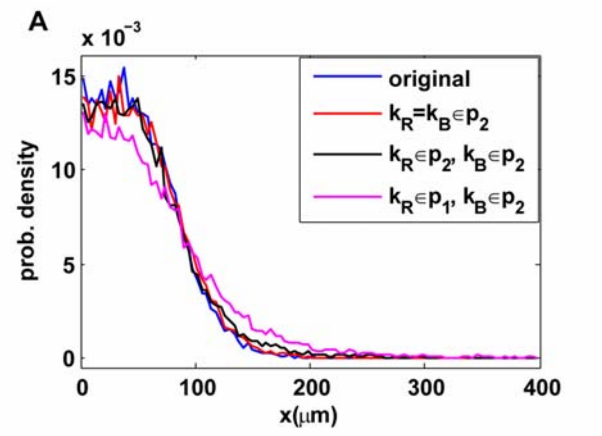

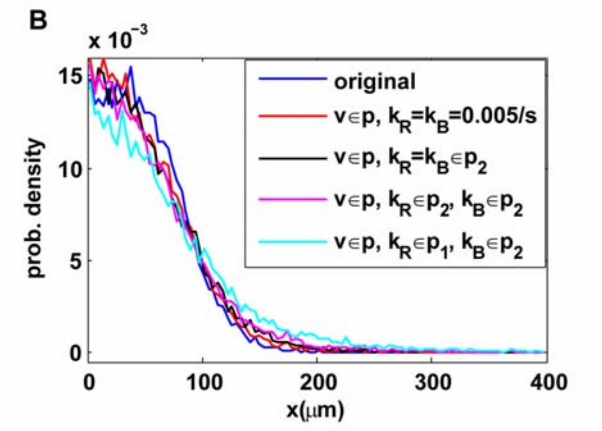

Figure S4 Effects of cell-to-cell variability in channel with linear gradient: cell number probability density along the channel with linear attractant gradient for populations of cells with variability in different parameters. A) Variability in adaptation rate. Original in the legend means no cell-cell variations, i.e., we use the same parameters for all the cells. We have checked three other conditions where methylation and demethylation rate constants are chosen randomly from the normal distributions $p_1(k_{R(B)}) = \frac{1}{\sqrt{2}}\exp(-(\frac{k_{R(B)} - k_1}{\sigma})^2), p_2(k_{R(B)}) = \frac{1}{\sqrt{2}}\exp(-(\frac{k_{R(B)} - k_2}{\sigma})^2)$ with $k_1 = 0.01/s, k_2 = 0.005/s, \sigma = 0.002/s$. B) Variability in run velocity. Run velocity of individual cells are chosen from the Maxwell distribution $p(v) = \frac{2|v|}{v_0{}^2}\exp(-\frac{v^2}{v_0{}^2})$ with $v_0 \approx 20\mu m/s$. At the population level, the average steady state behavior remains mostly the same except for small changes near the boundary.
